# High-resolution mapping of a major effect QTL from wild tomato *Solanum habrochaites* that influences water relations under root chilling

**DOI:** 10.1007/s00122-015-2540-y

**Published:** 2015-06-05

**Authors:** Erin M. Arms, Arnold J. Bloom, Dina A. St. Clair

**Affiliations:** Plant Sciences Department, University of California-Davis, Mail Stop 3, Davis, CA 95616 USA

## Abstract

*****Key message***:**

**QTL*stm9*controlling rapid-onset water stress tolerance in*S. habrochaites*was high-resolution mapped to a chromosome 9 region that contains genes associated with abiotic stress tolerances.**

**Abstract:**

Wild tomato (*Solanum habrochaites*) exhibits tolerance to abiotic stresses, including drought and chilling. Root chilling (6 °C) induces rapid-onset water stress by impeding water movement from roots to shoots. *S. habrochaites* responds to such changes by closing stomata and maintaining shoot turgor, while cultivated tomato (*S. lycopersicum*) fails to close stomata and wilts. This response (shoot turgor maintenance under root chilling) is controlled by a major QTL (designated *stm9*) on chromosome 9, which was previously fine-mapped to a 2.7-cM region. Recombinant sub-near-isogenic lines for chromosome 9 were marker-selected, phenotyped for shoot turgor maintenance under root chilling in two sets of replicated experiments (Fall and Spring), and the data were used to high-resolution map QTL *stm9* to a 0.32-cM region. QTL mapping revealed a single QTL that was coincident for both the Spring and Fall datasets, suggesting that the gene or genes contributing to shoot turgor maintenance under root chilling reside within the marker interval H9–T1673. In the *S. lycopersicum* reference genome sequence, this chromosome 9 region is gene-rich and contains representatives of gene families that have been associated with abiotic stress tolerance.

**Electronic supplementary material:**

The online version of this article (doi:10.1007/s00122-015-2540-y) contains supplementary material, which is available to authorized users.

## Introduction

Abiotic stresses, especially those which affect the water relations of the plant such as low temperatures, may decrease plant growth and yield. The majority of plants will suffer damage when exposed to freezing temperatures (those below 0 °C), but plants of tropical or sub-tropical origin also suffer damage when exposed to chilling temperatures (i.e., above 0 and below 10 °C) (Catala and Salinas 2010; Geisenberg and Stewart 1986). Exposure of roots to chilling temperatures decreases root hydraulic conductance (Aroca et al. 2001; Vernieri et al. 2001), and can result in water stress and chilling injury within a few hours of exposure (Aroca et al. 2012, 2001; Bloom et al. 2004). The susceptibility to water stress induced by root chilling in species of tropical and sub-tropical origin is a concern for agricultural production in Mediterranean climates such as California, where exposure to cold soils in the spring can affect seedling establishment because soil temperatures under an open canopy may be colder than air temperatures (Nobel 1999).

Cultivated tomato (*Solanum lycopersicum*) is a classic example of a chilling-sensitive crop (Geisenberg and Stewart 1986). It was domesticated from the wild cherry tomato, which is native to mesic, tropical environments (Rick 1983). A related wild tomato species, *S. habrochaites*, grows in the Peruvian Andes at altitudes up to 3300 m and thrives in xeric habitats and at chilling temperatures detrimental to *S. lycopersicum* (Jung et al. 1998; Vallejos and Pearcy 1987; Venema et al. 1999). Upon exposure to root chilling conditions, the root hydraulic conductance of both tomato species decreases, but *S. habrochaites* closes its stomata rapidly in response to chilling stress, thereby maintaining water potential and shoot turgor, whereas the stomata of *S. lycopersicum* remains open and the shoots wilt (Bloom et al. 2004). Other agronomically important crops of tropical or sub-tropical origin such as maize and rice respond to root chilling in a manner consistent with that of cultivated tomato (Aroca et al. 2001; Cruz et al. 2013). An improved understanding of the underlying mechanisms of root chilling tolerance in wild *S. habrochaites* would contribute to a better general understanding of chilling sensitivity in crops of tropical and sub-tropical origins.

There are few published studies on the genetics of tolerance to chilling temperatures in tomato. A review by Venema et al. (2005) focused on physiological effects of chilling and noted that wild tomato species were promising sources of genetic tolerance to chilling. Oyanedel et al. (2001) evaluated a backcross inbred line population derived from *S. habrochaites* acc. LA1777 for growth traits under chilling temperatures and reported QTL for higher biomass accumulation on chromosomes 2, 3, and 9. Elizondo and Oyanedel (2010) evaluated tomato introgression lines (ILs) containing *S. habrochaites* acc. LA1777 introgressions on chromosomes 2 and 3 in the field under low temperatures (below 12 °C). The ILs had higher growth rates but lower fruit set than the parental lines in response to an increase in the number of hours of chilling temperatures.

To investigate the genetic basis of shoot turgor maintenance under root chilling, Truco et al. (2000) used an interspecific BC_1_ population derived from chilling-susceptible *S. lycopersicum* cv. T5 and chilling-tolerant wild *S. habrochaites* acc. LA1778 to map QTL for this trait. Three QTL for shoot turgor maintenance under root chilling were identified on chromosomes 5, 6, and 9. The largest effect QTL located on chromosome 9 accounted for 33 % of the trait phenotypic variance (Truco et al. 2000). We designated this QTL *stm9* for shoot turgor maintenance, chromosome 9. Subsequently, QTL *stm9* was fine-mapped to a 2.7-cM region on the short arm of chromosome 9 between markers T1670 and T1673 (Goodstal et al. 2005). Easlon et al. (2013) determined that tomato ILs containing the short arm of chromosome 9 from chilling-tolerant *S. lycopersicoides* and *S. habrochaites* maintained shoot turgor under root chilling.

Here we high-resolution mapped QTL *stm9* using recombinant sub-near-isogenic lines and compared high-resolution mapped QTL *stm9* to the *S. lycopersicum* reference genome for initial identification of potential candidate genes and regulatory sequences (assembled *S. habrochaites* whole genome sequence is not available). Our longer term goal is to identify and functionally test candidate genes and regulatory sequences from *S. habrochaites* and determine the causal gene(s) or polymorphisms for QTL *stm9*.

## Materials and methods

### Plant material

**Fig. 1:**
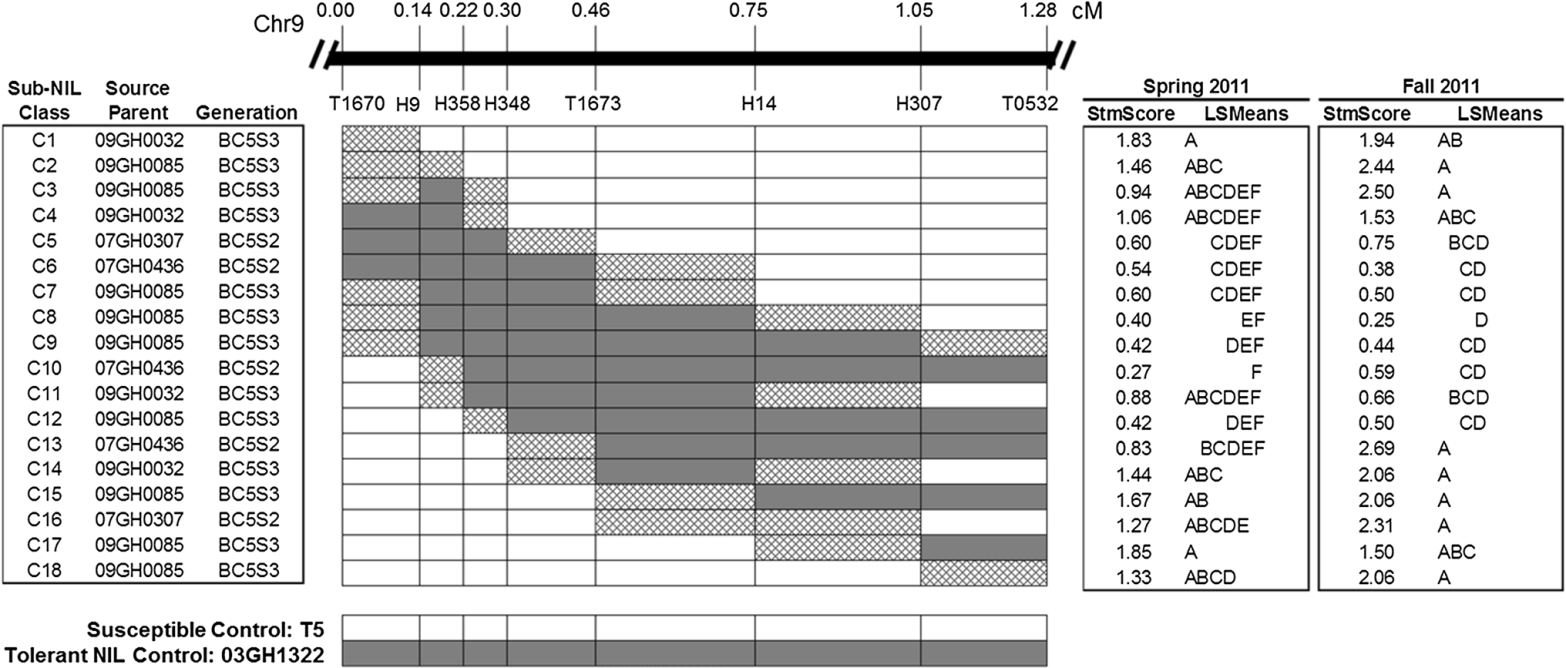
Graphical marker genotypes and stmscore least square (LS) means of chromosome 9 sub-NILs used to high-resolution map QTL *stm9* from *S. habrochaites.* Replicated experiments to phenotype sub-NILs and controls for stmscore were conducted in a greenhouse during Spring and Fall 2011. Sub-NIL recombinant genotypic classes (C1–C18), source parent identity, and generation for each of the 18 sub-NILs used in this study are shown. Linkage map (in cM) for the introgressed *S. habrochaites* region is displayed above the graphical genotypes for sub-NILs and control genotypes. Homozygous *S. habrochaites* regions are solid gray, recombination breakpoint regions are crosshatched, and homozygous *S. lycopersicum* regions are white. To the right of the figure are least square (LS) means groupings based on Tukey’s HSD test for Spring and Fall 2011 datasets

A population of near-isogenic lines (NILs) containing the chromosome 9 region (including QTL *stm9*) from *S. habrochaites* acc. LA1778 in an otherwise completely *S. lycopersicum* cv. T5 background was marker-selected and used for fine-mapping, as described in Goodstal et al. (2005). For high-resolution mapping of *stm9*, we created and marker-selected recombinant sub-near-isogenic lines (sub-NILs) as follows. The heterozygous BC_4_ line 04GH0030 from Goodstal et al. (2005) containing a single copy of the *S. habrochaites* allele for fine-mapped QTL *stm9* was used to generate a BC_5_ segregating for only the chromosome 9 region of interest from *S. habrochaites*. The BC_5_S_1_ generation was marker genotyped to identify recombinant individuals within the fine-mapped *stm9* region flanked by markers T1670 and T0532 (Fig. 1). Self-pollinated seed was collected from individuals chosen for their chromosome 9 introgressions, and these BC_5_S_1_ individuals were marker-screened for further recombination events within the chromosome interval containing *stm9*.

Self-seed from two heterozygous BC_5_S_1_ individuals (07GH0436 and 07GH0307) and two heterozygous BC_5_S_2_ individuals (09GH0085 and 09GH0032) were screened for recombinants via marker-assisted selection (MAS) (see *MAS for Identification of Recombinants*, below). Individuals that contained recombination events within the chromosome 9 fine-mapped *stm9* region were selected, grown to maturity, and allowed to self-pollinate to produce seed of fully homozygous individual sub-NILs in the BC_5_S_2_ or BC_5_S_3_ generation (as determined by the generation of their original parent line, 09GH0085 and 09GH0032, respectively). Recombinant homozygous individual sub-NILs were allowed to self-pollinate to generate ample seeds for replicated experiments. Phenotyping experiments were performed with one representative line from each recombinant class.

All plant materials were grown in greenhouses at UC Davis. Seeds were planted in 73-cell flats containing soil media. Flats were watered daily, and plants were fertilized with a 10:30:20 NPK solution once a week. Greenhouses containing plants in flats, pots, and hydroponic tanks were maintained at ambient conditions of 25–37 °C with 55–80 % relative humidity during the day, and 18–25 °C with 20–55 % relative humidity at night. Plants from which seed was to be collected were transferred at the 4th to 5th true leaf stage (approximately 2 months post-germination) to individual 8-L pots filled with soil media, and grown to maturity to obtain seed.

### Development of SNP-based markers for MAS

SNP markers were identified specifically for our project or converted from publicly available *S. lycopersicum* markers (Bombarely et al. 2011; SGN solgenomics.net). Three markers used by Goodstal et al. (2005) were included: two PCR-based Cleaved Amplified Polymorphic (CAP) markers, T1670 and T0532, and one Sequence Characterized Amplified Region (SCAR) marker, T1673. We used two additional markers (TG18 and At5g11560) from the Tomato-EXPEN 2000 linkage map (SGN solgenomics.net). To convert the above-listed markers to SNP markers, DNA from *S. lycopersicum* cv. T5 and the interspecific F_1_ hybrid (*S. lycopersicum* cv. T5 × *S. habrochaites* acc. LA1778) was amplified using Phusion High-Fidelity DNA Polymerase (New England Biolabs, Inc., Ipswich, MA, USA) with recommended reagents and cycling conditions (30 s at 98 °C, followed by 35 cycles of 10 s at 98 °C, 20 s at 55 °C, 15 s at 72 °C, then 5 min at 72 °C). PCR products were sequenced by the UC Davis CBS DNA Sequencing Facility using Sanger sequencing on an ABI 3730 Capillary Electrophoresis Genetic Analyzer. PCR product sequences from *S. lycopersicum* cv. T5 and the interspecific F_1_ hybrid were compared for each marker set using CLC Sequence Viewer 6 (CLC Bio, Cambridge, MA, USA http://www.clcbio.com) to identify SNPs (Online Resource 1).

During the course of our marker development, a pre-publication version of the cultivated tomato (*S*. *lycopersicum*) reference genome sequence became available. Therefore, we designed additional markers from this sequence (Tomato WGS Scaffolds v1.00; SGN solgenomics.net). The markers obtained from Goodstal et al. (2005) and the Tomato-EXPEN 2000 linkage map were mapped to the scaffold sequence using the BLAST tool on the Sol Genomics Network website (SGN solgenomics.net), and new primers were designed in regions between the mapped markers. The new primers were used to amplify and sequence the targeted regions between the BLASTed markers. The sequences obtained were aligned as described previously. Of nine primer sets tested, two (designated H9 and H14) amplified consistently and exhibited polymorphisms suitable for the development of SNP markers (Online Resource 1).

As the preliminary genomic annotation provided by the International Tomato Annotation Group (ITAG) (Consortium 2012; SGN solgenomics.net) became publicly available, predicted gene sequences in our target region were BLASTed against the latest version of the genome sequence to identify single copy genes. Primers were designed for those single copy genes that mapped to scaffolds of the chromosome 9 region containing fine-mapped QTL *stm9.* Scaffolds containing QTL *stm9* were identified using the markers that were already developed for our project. Three gene sequences amplified consistently and had polymorphisms suitable for the development of SNP markers. All three predicted genes mapped to scaffold 06070 of ITAG version 1.00 (chromosome 9 in SL2.50) (SGN solgenomics.net). The SNP markers developed from these predicted gene sequences were numbered according to the gene model from which they were designed: H348, H358, and H307 (Online Resource 1).

Using the markers we developed for chromosome 9, a multiplexed SNP genotyping assay was designed using Sequenom’s MassARRAY Assay Design 3.1 Software. Software presets for Single Base Extension (SBE) High-Multiplexing were used, with one SNP per marker for TG18, T1670, and T1673 and two SNPs per marker for T0532, At5g11560, H9, and H14. As additional markers were developed, they were added using the software’s Superplex Replex mode for a multiplex assay with an additional SNP for H358 and two SNPs for H348 (Online Resource 1).

### MAS for identification of recombinants for high-resolution mapping of *stm9*

DNA of each seedling screened for potential recombination events was extracted from two young leaflets using a 96-well format CTAB procedure (Fulton et al. 1995). DNA extracts were diluted to 5–20 ng/µL, and 1 µL of the diluted DNA was used as the template for Sequenom’s MassARRAY i-Plex Gold genotyping. Samples were processed according to Sequenom’s i-Plex Gold Application guide with the following modifications: PCR settings for amplifying DNA for genotyping were: 95 °C for 2 min; 45 cycles of 95 °C for 30 s, 56 °C for 30 s, 72 °C for 1 min; followed by 72 °C for 5 min and then hold at 10 °C. PCR settings for the i-Plex extension reaction were: 94 °C for 2 min; 40 cycles of 94 °C for 5 s, 52 °C for 5 s, 80 °C for 3 s, 52 °C for 5 s, 80 °C for 3 s, 52 °C for 5 s, 80 °C for 3 s, 52 °C for 5 s, 80 °C for 3 s, 52 °C for 5 s, 80 °C for 3 s; followed by 72 °C for 3 min and then hold at 10 °C. After i-Plex extension and product clean-up, plates were submitted to the UC Davis Veterinary Genetics Lab where they were run on a MassARRAY Analyzer Compact to obtain genotypic data.

### Phenotyping of recombinant sub-NILs

After 2 weeks of growth in flats containing soil media (see *Plant Material*, above), the roots of two seedlings of each sub-NIL or control were carefully washed free of soil media in deionized water and transferred to a hydroponic growth tank set at 20 °C containing a modified Hoagland solution at 20 % of full strength (Epstein and Bloom 2005). Efforts were made to use vigorous seedlings of similar size. Plants were grown in the hydroponic tank for 1 week under ambient illumination in the greenhouse, with constant aeration and circulation of the nutrient solution. Subsequently, the plants were randomized (see next paragraph) and transferred to a separate refrigerated hydroponic tank containing fresh modified Hoagland solution at 20 % strength. Plants were acclimated overnight at a solution temperature of 20 °C. The following morning supplementary lighting was provided by one 1000 W metal halide lamp starting at 7:00 am and used throughout the experiment to maintain a light level above 1000 µmol m^−2^ s^−2^ PAR. The tank solution was maintained at 20 °C for 1 h after the supplementary lighting was turned on, and then the tank temperature was decreased to 6 °C. The tank was held at 6 °C for 2 h prior to phenotyping.

Each experiment was conducted as a Randomized Complete Block Design (RCBD) and repeated in two seasons (Spring and Fall of 2011), with days as blocks and two replicate plants of each genotype per block. In addition to the two individual plants per recombinant sub-NIL, each replication of the experiment included controls: four plants of chilling-sensitive *S. lycopersicum* cv. T5, and two plants of a chilling-tolerant NIL, 03GH1322 (designated hereafter as NIL1322) that was also used as a tolerant control by Goodstal et al. (2005). Six repetitions of the experiment that were conducted from May 9th to June 13th comprise the Spring data set, and four repetitions of the experiment that were carried out from October 7th to October 20th comprise the Fall data set. Plants were individually phenotyped for shoot turgor maintenance under root chilling (stmscore) according to the rating scale described in Goodstal et al. (2005). Briefly, shoot turgor was scored for each plant on a scale of 0–3, with a stmscore of 0 denoting maintenance of shoot turgor, and a stmscore of 3 denoting severe loss of shoot turgor (flaccid).

### Statistical analysis

Prior to conducting analysis of variance (ANOVA), stmscore trait data were subjected to the Shapiro–Wilk test for normality and the Levene’s test for homogeneity of variances using PROC GLM in SAS v9.3 (SAS Institute, Cary, NC, USA). The assumption of normality was met when Shapiro–Wilk *W* > 0.95. The Levene’s test was considered significant for a factor in the linear additive model (LAM) when *P* ≤ 0.05. A significant Levene’s was indicative of heterogeneity of variances (HOV), in which case data were weighted by the inverse of the variance for the significant factor. The data were analyzed as an RCBD, with Days as the blocking term. ANOVA of stmscore was performed with the mixed linear model procedure (PROC MIXED) in SAS using the following LAM:

$$ {\text{Stmscore}} = {\text{Season}} + {\text{Genotype}} + {\text{Genotype}}*{\text{Season}} $$

where Season was the season (Spring or Fall), and Genotype * Season denotes an interaction. Day (Season), which designates Day nested within Season, was specified in the random statement, where Day refers to the replications of the experiment (blocking factor). All other factors were considered fixed. Due to a significant Genotype * Season interaction (*P* ≤ 0.05), each season was analyzed separately with the following LAM:

$$ {\text{Stmscore}} = {\text{Genotype}} $$

where Day was considered a random factor and included in the random statement. Because HOV for genotype was significant, stmscore was weighted by the inverse of the variance associated with Genotype to meet the assumptions of ANOVA. Tukey’s Honestly Significant Difference (HSD) test was used to perform stmscore mean separations for each season using the least square means (LSMeans) statement in PROC MIXED. The PDMIX800 macro was used to obtain letter groupings as part of the SAS output (Saxton 1998).

### Linkage and QTL mapping

The linkage map for the *S. habrochaites* introgressed chromosome 9 region was constructed with JoinMap 4.0 (Van Ooijen 2006). The Kosambi function with a 4-LOD significance threshold was used to construct the map; the resulting marker grouping was maintained at LOD 10. The population used for map construction included all 2862 BC_5_S_2_ and BC_5_S_3_ individuals that were genotyped (see previous section on *MAS for Identification of Recombinants*). Since no recombinants were identified between markers T1670 and TG18, or between markers H348 and At5g11560, markers TG18 and At5g11560 were not included in subsequent analyses.

QTL mapping of stmscore was conducted for each season using sub-NIL LSmeans obtained from ANOVA. QTL mapping was performed with WinQTLCartographer2.5 (Wang et al. 2011) using composite interval mapping (CIM) Model 6 (standard model) with forward and backward regression. Due to the relatively small genetic distances between markers, a walk speed of 0.5 cM and a window size of 0.5 cM were used. One thousand permutations were performed to obtain a trait-specific permuted significance threshold at *P* = 0.05; a significant QTL was declared when the LOD value exceeded the permuted threshold.

### Alignment of the high-resolution mapped QTL stm9 region with the *S. lycopersicum* reference genome sequence

We used the publicly available *S. lycopersicum* reference genome sequence version SL2.50 to obtain estimates of physical size and gene content in the QTL *stm9* region because assembled *S. habrochaites* whole genome sequence is not available. The *S. habrochaites* genome is 1.5 × the size of the *S. lycopersicum* genome as determined by flow cytometry (Arms and St.Clair, unpublished data). The location of the QTL *stm9* region in *S. lycopersicum* was determined on the Sol Genomics Network *S. lycopersicum* reference genome version SL2.50 browser using BLAST (SGN solgenomics.net). The BLAST position of the midpoint of each *S. lycopersicum* cv. T5 sequence that we obtained from PCR product sequencing during SNP marker development was defined as the physical location of that marker in the *S. lycopersicum* reference genome SL2.50. The physical positions of the markers on SL2.50 were used to compare the *S. lycopersicum* physical map to the genetic map for QTL *stm9*. Kilobases (kbp) per 1 cM were calculated for the QTL *stm9* region from the flanking markers H9 to T1673, as well as for each internal marker-to-marker interval (Fig. 3).

Once the physical size of the QTL *stm9* region in *S. lycopersicum* was estimated, the number and identity of annotated genes were obtained from ITAG (International Tomato Annotation Group) release 2.40 (SGN solgenomics.net). Gene name, location, protein sequence analysis, and classification information InterPro (IPRO) and Gene Ontology (GO) annotations were downloaded from the SGN genome browser Gene Track. Genes were considered within the QTL region if any exonic sequence of a given gene fell within the QTL consensus region identified in both the Spring and Fall datasets, and defined by the flanking marker interval H9 to T1673. Genes were categorized according to function and/or type when GO terms and IPRO definitions were available (Online Resource 2).

## Results

### Significant differences among recombinant sub-NILs for shoot response to root chilling

From a total of 2862 BC_5_S_2_ and BC_s_S_3_ individuals genotyped, 52 individual recombinant sub-NILs that represented 18 unique recombinant classes (Fig. 1) were identified, with at least two independent recombinant sub-NILs identified in each recombinant class. Recombinant sub-NILs (designated C1–C18, see Fig. 1) were subjected to replicated experiments in hydroponic tanks, and stmscore data were obtained for Fall and Spring. A full model ANOVA of stmscore detected a highly significant Genotype × Season interaction (*P* < 0.0001), therefore separate ANOVAs for the Spring and Fall 2011 data sets were performed (Table 1). In both seasons, Levene’s test was significant for Genotype. Consequently, the data were weighted by the reciprocal of the variance for Genotype to meet the assumptions of homogeneity of variance, and Proc MIXED was used to analyze each data set separately. Genotype was highly significant in both seasons (*P* < 0.0001) (Table 1).

**Table 1:** Summary of ANOVAs for stmscore (shoot turgor maintenance under root chilling) obtained from hydroponic tank experiments conducted with recombinant sub-NILs C1–C18 for the chromosome 9 region containing QTL *stm9*. Experiments were conducted during the Spring and Fall of 2011 in a greenhouse at UC Davis. Full model *F* values for the main effects were obtained from PROC MIXED in SAS. Due to the significant Genotype × Season interaction, an ANOVA using PROC MIXED was conducted independently for the Spring and Fall datasets. Linear additive models for the Full model and Spring/Fall datasets are given in “Materials and methods”

Linear additive model	Source of variation	*df* numerator	*df* denominator	*F* value
Full model	Season	1	8	12.33**
	Genotype	19	352	18.37***
	Genotype * Season	19	352	3.92***
Spring	Genotype	19	215	7.93***
Fall	Genotype	19	215	12.75***

* *P* ≤ 0.05; ** *P* ≤ 0.01; *** *P* ≤ 0.001

**Fig. 2:**
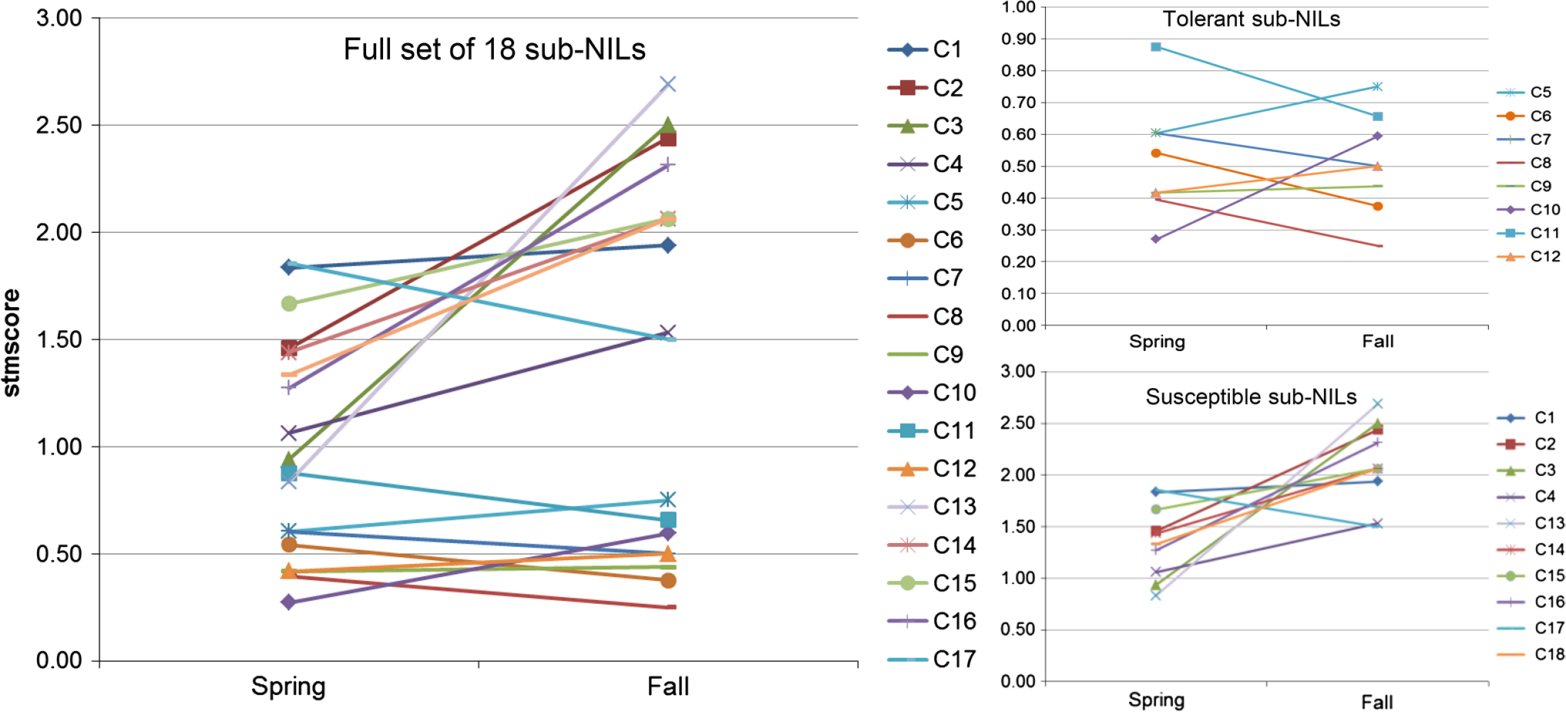
Interaction Plots for the Full Set (*left*), Tolerant (*upper right*), and Susceptible (*lower right*) chromosome 9 sub-NILs that were phenotyped for stmscore under root chilling. The *x*-axis is Season, and the *y*-axis is mean stmscore per sub-NIL. Shoot turgor was scored on a scale of 0–3, with a stmscore of 0 denoting maintenance of shoot turgor, and a stmscore of 3 denoting severe loss of shoot turgor (flaccid). Entries C1–C18 refer to sub-NIL graphical genotype designations (see Fig. 1)

Within each data set, genotype means were significantly different and several groupings of means were identified (Figs. 1, 2). Recombinant sub-NILs (C1–C18, Fig. 1) were classified into two main groups: susceptible or tolerant, according to a mean stmscore less than 1.0 in the Spring and Fall (tolerant) or mean stmscore greater than or equal to 1.0 (susceptible), respectively (Fig. 2). The Fall data set resulted in distinct groupings between susceptible and tolerant sub-NILs, with no overlap between the two groups (Fig. 2). The Spring data set exhibited a more gradual separation of means, with three sub-NILs (C3, C11, and C13) with a mean stmscore of just under 1.0, and one sub-NIL (C4) with a mean stmscore just over 1.0 (Figs. 1, 2).

While sub-NIL rank changes within the tolerant and susceptible groups occurred between the two data sets, rank changes did not result in the reassignment of any sub-NIL between the susceptible and tolerant groups, with the exception of sub-NILs C3 and C13. Sub-NILs C3 and C13 were grouped as tolerant in the Spring dataset (mean stmscore less than 1.0), and susceptible in the Fall dataset (mean stmscore greater than 1.0). Not only did these lines score as susceptible in the Fall dataset, they had the highest (most susceptible) mean stmscore of any recombinant sub-NIL in the Fall dataset, and therefore were designated as susceptible (Fig. 2).

### High-resolution mapping of QTL *stm9*

**Fig. 3:**
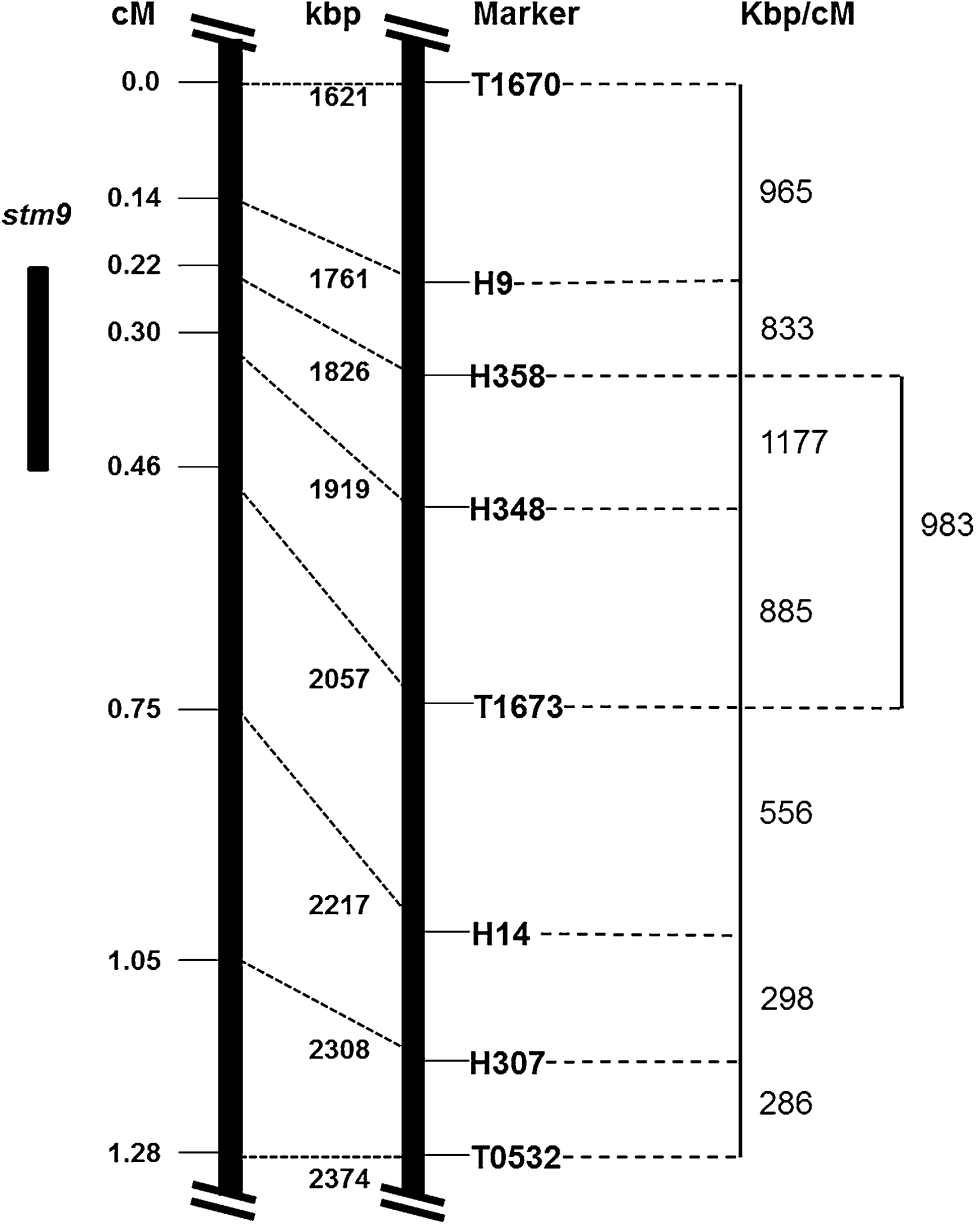
Comparison of the genetic map of chromosome 9 region containing QTL *stm9* with the physical map of the corresponding syntenic region from the *S. lycopersicum* reference genome SL2.50 (SGN solgenomics.net). On the left is the genetic map in cM; in the middle is the physical map of *S. lycopersicum* in kbp; and to the right is kbp/cM for high-resolution mapped *stm9* (marker interval H358–T1673), and for each interval between markers. High-resolution mapped QTL *stm9* is depicted to the left of the genetic map

The linkage map of the introgressed *S. habrochaites* chromosome 9 region included eight polymorphic markers that spanned 1.28 cM (Fig. 3). The average distance between markers was 0.18 cM, with the largest interval (0.29 cM) between markers T1673 and H14, and the smallest (0.08 cM) between markers H9–H358 and H358–H348.

**Fig. 4:**
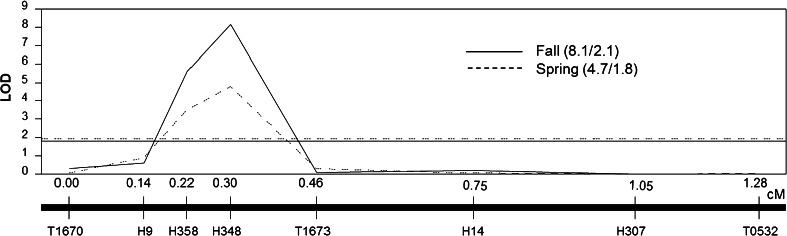
High-resolution mapped QTL *stm9* located on chromosome 9. Stmscore data obtained from replicated greenhouse experiments conducted during Spring and Fall 2011 with sub-NILs containing chromosome 9 introgressions from *S. habrochaites*. QTL analysis was conducted with CIM in QTLCartographer. The *y*-axis is LOD value, *x*-axis is genetic distance (in cM) within the introgressed region. Genetic markers are indicated below the solid black bar. LOD trace and threshold are denoted by a dashed line for Spring and solid line for Fall. LOD values above the threshold indicate significant QTL (at *P* = 0.05). Values for LOD threshold and peak LOD are displayed as (Peak/Threshold)

A single significant QTL was detected between markers H9 and T1673 with both the Spring and Fall datasets (Fig. 4). The QTL LOD peak was at marker H348 for both datasets. The 1-LOD and 2-LOD intervals for both data sets were defined by markers H358–T1673, with the exception of the 2-LOD interval for Spring. In this case the left-most bracketing marker was H9, not H358. There was no evidence of additional significant QTL or of fractionation of QTL *stm9* as a consequence of higher-resolution mapping. The QTL peak marker, H348, was also the only marker with the *S. habrochaites* allele across all recombinant sub-NILs consistently classified as tolerant (Figs. 1, 2). Furthermore, sub-NILs with the *S. habrochaites* allele at marker H348 and at one of the flanking markers (H358 or T1673) scored as tolerant. Our results strongly support the close linkage of the *S. habrochaites* gene(s) or polymorphisms responsible for maintenance of shoot turgor under root chilling to marker H348.

### Gene content in the *S. lycopersicum* chromosome 9 region syntenic to high-resolution mapped QTL *stm9*

The high-resolution mapped QTL *stm9* region corresponds to a physical distance of ~231 kb between markers H358 and T1673 on the short arm of chromosome 9 in the *S. lycopersicum* reference genome SL2.50 (Fig. 3). This region in *S. lycopersicum* contains 22 annotated genes (Online Resource 2), including several transcription factors, transporters, and regulatory elements (SGN solgenomics.net). Considering the genes located within the *S. lycopersicum* QTL *stm9* region, the genes most closely linked to the peak marker H348 (within ± 30 kb) are the following: Solyc09g8440.2.1 (C_2_H_2_ Zinc finger transcription factor), Solyc09g8450.2.1 (cytoskeleton structure), Solyc09g8460.2.1 (Ras-related protein/signal transduction), Solyc09g8470.2.1 (C_2_H_2_ Zinc finger transcription factor), Solyc09g8480.2.1 (stress-induced plant growth coordinator), and Solyc09g8490.2.1 (HEAT repeat family, intracellular transporter). The peak marker falls within an intronic region of Solyc09g8470.2.1.

## Discussion

### Genetic and environmental factors affecting the stmscore phenotype

The chromosomal location of *stm9* in our study agrees with Goodstal et al. (2005) who fine-mapped *stm9* to marker interval T1670–T1673 (~2.7 cM). We refined the location of *stm9* to marker interval H358–T1673, a genetic distance of 0.32 cM. Our data suggests that the gene(s) or polymorphisms controlling the tolerance phenotype are located close to marker H348 and within the marker interval H358–T1673. The chromosomal location of QTL *stm9* detected in both data sets was coincident despite the significant Genotype × Season interaction in the ANOVA. To examine the cause of the significant Genotype × Season interaction in more detail, we plotted recombinant sub-NIL stmscore means across the two seasons to create interaction plots (Fig. 2). Inspection of the plots suggests that the changes in sub-NIL mean values across seasons primarily derived from greater chilling susceptibility (mean stmscore ≥1.0) of susceptible sub-NILs in the Spring than in the Fall (Fig. 2). Magnitude differences would cause the size of the LOD peaks to differ among seasons, but not change the peak location, which is in agreement with our results (Fig. 4).

In addition to the increase in the magnitude of means for stmscore of the susceptible sub-NILs, two sub-NILs (C3 and C13) were classified as tolerant (mean stmscore less than 1.0) in the Spring dataset but as susceptible (mean stmscore ≥1.0) in the Fall dataset (Fig. 2). Sub-NIL C4 had a mean of slightly over 1.0 in the Spring data set, and clearly grouped as susceptible in the Fall dataset (Fig. 2). None of these lines contain the *S. habrochaites* introgression for high-resolution mapped *stm9*, but the introgressions do all flank *stm9*. These results suggest the possibility that there are environmentally sensitive genetic modifiers of the stmscore phenotype in this region of chromosome 9, and that the interaction of these modifiers with the environment could be causing the significant Genotype × Season interaction. Phenotypic plasticity (i.e., the interaction of genotype by environment due to variable environmental cues) in the presence of abiotic stress has been noted and reviewed previously (Juenger 2013; Kleunen and Fischer 2005). The rank changes seen within the tolerant group may be due to differences in the genomic content of *S. habrochaites* sequence in the flanking regions of QTL *stm9*, and not a direct effect of the environment on the gene(s) or polymorphisms controlling the tolerant *stm9* phenotype.

Previous work in tomato has shown that the stomatal response of a plant when subjected to root chilling conditions differs between susceptible and tolerant phenotypes (Bloom et al. 2004). Stomatal control is regulated by multiple environmental factors including light, temperature, day length, humidity, and CO_2_ levels (Assmann and Wang 2001; Chaves et al. 2003; Damour et al. 2010; Roelfsema and Hedrich 2005). The Spring experiments were conducted under longer day lengths, higher air temperatures, and lower humidity than the Fall experiments (LAWR 2013). These seasonal differences affect the conditions in the greenhouse and may have contributed to the significant Genotype × Season interaction, as well as differences in relative response among the sub-NILs in the Spring versus Fall data sets. In the context of phenotypic plasticity, seasonal effects on sub-NIL performance would account for the more gradual separation of means in the Spring dataset compared to the Fall (Figs. 1, 2) (Juenger 2013; Kleunen and Fischer 2005).

### QTL mapping precision and resolution

Low marker density and small population sizes in initial genome-wide QTL mapping studies may bias upwards the estimation of QTL effects due to the inability to resolve closely linked, smaller effect QTL (Mackay et al. 2009). Consequently, single large effect QTL may resolve or fractionate into multiple, smaller effect QTL after fine- and high-resolution mapping (Haggard et al. 2013; Johnson et al. 2012; Studer and Doebley 2011). The original interspecific BC_1_ population used by Truco et al. (2000) to map QTL for shoot turgor maintenance under root chilling consisted of 196 individuals genotyped with 112 markers. Truco et al. (2000) mapped a major effect QTL (later designated as *stm9* by Goodstal et al. 2005) to a 28-cM region on the short arm of chromosome 9 which accounted for 33 % of the phenotypic variation for shoot turgor maintenance under root chilling (Truco et al. 2000). Despite the large initial genetic size of the QTL *stm9* region detected by Truco et al. (2000), subsequent fine-mapping by Goodstal et al. (2005) and high-resolution mapping in our present study do not provide any evidence of multiple QTL or QTL fractionation.

The relatively small genetic size (0.32 cM) of high-resolution mapped *stm9* and the lack of QTL fractionation indicates that this level of resolution is suitable for the identification of candidate genes for *stm9*. There are numerous examples in the literature of environmentally stable, high-resolution mapped QTL that have led to candidate gene identification and in some cases subsequent causal gene/polymorphism determination. Several QTL for chilling tolerance in rice have been high-resolution mapped and candidate genes identified. These QTL include *qCTS12* (seedling chilling tolerance at 9 °C), *qCtss11* (seedling chilling tolerance at 4 °C), and *qCTB7* (cold tolerance during booting at 15 °C) (Andaya and Tai 2006; Koseki et al. 2010; Zhou et al. 2010). Tomato-specific QTL examples include *fw2.2,* a fruit weight QTL, and *se2.1*, a stigma exsertion QTL, both identified in progeny derived from *S. pennellii*, another wild tomato relative (Alpert and Tanksley 1996; Chen and Tanksley 2004). The causal gene underlying QTL *fw2.2* was identified by Frary et al. (2000), who proposed that changes in the regulation of ORFX (an unidentified open reading frame), not changes in the sequence or structure of the expressed protein, are responsible for changes in fruit size. Chen and Tanksley (2004) determined the casual mutation underlying *se2.1* is a mutation in the *Style2.1* promoter that results in a down-regulation of *Style2.1* expression during flower development. Collectively, the results from these studies suggest that candidate gene identification and functional testing for QTL *stm9* should focus on mutations in regulatory and promoter regions of candidate genes in addition to mutations that may affect the sequence or structure of expressed proteins.

### Comparisons of high-resolution mapped QTL *stm9* region to the *S. lycopersicum* reference genome

Many genes have been identified as being involved directly or indirectly in plant tolerance or resistance to abiotic stresses (Cramer et al. 2011; dos Reis et al. 2012), including chilling/cold tolerance (Chinnusamy et al. 2006; Krasensky and Jonak 2012). Plant responses to abiotic stresses can include multiple pathways that involve a variety of gene products such as receptors, signaling molecules, transporters, transcription regulators, and transcription factors (Cramer et al. 2011; dos Reis et al. 2012). Many of the identified stress response pathways have been associated with tolerance to a range of abiotic stresses (Cramer et al. 2011; dos Reis et al. 2012; Krasensky and Jonak 2012). The plant’s response to abiotic stress may result in both reversible and irreversible activation of stress response pathways (Cramer et al. 2011). Because of the complex nature of the pathways involved, the specific genotype of the plant also has a large influence on abiotic stress response (Cramer et al. 2011; dos Reis et al. 2012). Plant responses to abiotic stressors are dependent on the interplay of abiotic stress, environment, and genotype (Cramer et al. 2011; dos Reis et al. 2012). Therefore, a particular abiotic stress applied in different environmental contexts may result in overlapping, but distinct responses from a single genotype (Cramer et al. 2011).

We analyzed the physical region in the cultivated tomato (*S. lycopersicum*) reference genome that is syntenic to the *S. habrochaites* QTL *stm9* region because an assembled *S. habrochaites* whole genome sequence is not available. All of the protein products of the *S. lycopersicum* annotated genes located within 30 kb of the QTL *stm9* peak marker (H358) have features that are shared with genes involved in responses to water stress and other abiotic stresses. In addition, the majority of the *S. lycopersicum* genes located within the syntenic high-resolution mapped *stm9* region have been implicated in abiotic stress response pathways (Assmann and Wang 2001; Ciftci-Yilmaz and Mittler 2008; Kiełbowicz-Matuk 2012; Nibau et al. 2006; Zhang et al. 2011) (Online Resource 2). It is possible that plant responses to root chilling stress may induce a more complex transcriptional response than other types of water stress such as those caused by salt or polyethylene-glycol (PEG), although overlap has been seen in the response to all three stresses (Tattersall et al. 2007). For example, in grape, under root chilling stress (5 °C) only transcripts for protein synthesis and the cell cycle were up-regulated to a lesser extent than under salt or PEG stress. The regulation of plant metabolism, protein metabolism, signal transduction, calcium signaling, stress hormone pathways, and transcription factors were all increased to a greater extent under root chilling in grape (Tattersall et al. 2007). These categories of genes account for the majority of genes located within the syntenic *S. lycopersicum* QTL *stm9* region.

While the total number of annotated genes (22) within the *S. lycopersicum* reference genome region containing QTL *stm9* is relatively small, there are no estimates available for *S. habrochaites* due to the unavailability of assembled whole genome sequence for this wild species. A comparison of the genetic and *S. lycopersicum* physical maps of the chromosome 9 region containing *stm9* shows a variable rate of recombination across this region (Fig. 3). The average kbp/cM for marker interval T1670–T1673 is 952 kbp/cM, whereas for marker interval T1673–T0532, it is 385 kbp/cM. Recombination occurs more frequently in gene-rich euchromatic regions, but can be suppressed due to lack of homology, heterochromatic regions, and/or the presence of repetitive elements (Henderson 2012; Korol 2013). It is possible that this variable rate of recombination is due to the presence of repetitive elements or other local structural polymorphisms affecting the synteny and colinearity of the *S. lycopersicum* and *S. habrochaites* genome sequences in this region. In addition, our flow cytometry results indicated that the genome size of *S. habrochaites* is 1.5 × that of *S. lycopersicum* (Arms and St.Clair, unpublished). The larger genome size of *S. habrochaites* suggests the possibility that the putative loss of function of genes and/or genetic elements in *S. lycopersicum* may be due to deletions or non-functional null mutations.

Matsuba et al. (2013) sequenced a functional gene cluster for terpene biosynthesis on chromosome 8 of *S. habrochaites* acc. 1778 and identified several rearrangements, deletions, and a novel gene when compared to the same gene cluster on chromosome 8 of the *S. lycopersicum* reference genome. Our prior research suggests that the inability of cultivated tomato to maintain shoot turgor under root chilling is the result of a loss of function in *S. lycopersicum* (Bloom et al. 2004; Goodstal et al. 2005). Taken together, the current evidence suggests that the *S. habrochaites* allele for high-resolution mapped QTL *stm9* may not be completely syntenic to *S. lycopersicum*, and that it may not contain the same genic compliment as the *S. lycopersicum* allele for *stm9*. Therefore, although the *S. lycopersicum* genome sequence is helpful in identifying potential candidate genes for shoot turgor maintenance under root chilling, the genomic sequence of the *stm9* region of *S. habrochaites* is necessary for accurate, well-informed candidate gene identification.

### Stability of QTL *stm9* and potential for use in breeding

Stability of QTL expression for tolerance to abiotic stresses is important for successful deployment of stress tolerance QTL in breeding crop plants. Although a significant Genotype × Season interaction was identified for QTL *stm9*, the potential causes of the interaction suggest that this region would likely be useful as a stable source of root chilling tolerance for breeding. A number of other QTL have been identified as targets for breeding despite a significant Genotype × Season interaction in several species, including barley, rice, and maize (Jompuk et al. 2005; Kalladan et al. 2013; Yadaw et al. 2013). The phenotypic plasticity likely contributed by the *stm9* flanking regions suggest that any future breeding strategies should be undertaken with the smallest introgression possible that still contains the entire high-resolution mapped QTL *stm9.* The *S. habrochaites* introgression in sub-NIL C7 contains only the high-resolution QTL *stm9* region (marker interval H9–T1673). This sub-NIL was grouped as tolerant in both the Spring and Fall datasets, and gave a consistently low (i.e., tolerant) stmscore in both seasons (Fig. 2), suggesting it may serve as a suitable potential donor parent source of tolerance to root chilling in breeding programs.

Due to the complexity of the abiotic stress response pathway, it is unlikely that the *S. habrochaites* QTL *stm9* allele contains only a single gene conferring shoot turgor maintenance under root chilling. Single causal genes have been identified for a number of major QTL (Chen et al. 2007; Frary et al. 2000; Liu et al. 2002; Uauy et al. 2006), but other major QTL have been shown to be controlled by two or more causal genes or polymorphisms (Chen and Tanksley 2004; Xu et al. 2006). Identification and testing of the causal gene(s) or polymorphisms underlying QTL *stm9* for tolerance to root chilling will be an important step in the identification of genetic targets for improving stress tolerance of plants exposed to root chilling and other types of water stress through marker-assisted breeding. Determination of the gene(s)/polymorphisms responsible for a quantitative trait phenotype is facilitated by genomic approaches (Collins et al. 2008; Flint and Mott 2001; Tanksley and Fulton 2007). Once a target region is identified via high-resolution mapping, a combination of genomic sequencing, structural genomic analysis, and transcriptome profiling can be used to assist in the identification of candidate genes. Therefore a biologically informed ranking of candidate genes located within the QTL *stm9* region will require a combination of *S. habrochaites* genome sequence for this region as well as transcription profiles for susceptible and tolerant sub-NILs exposed to root chilling. It is hoped that a better understanding of the underlying mechanism for tolerance to rapid-onset water stress in wild tomato *S. habrochaites* may aid in the identification of chilling tolerance genes in other species of tropical and sub-tropical origin.

#### Author contribution statement

DS and AB conceived and designed the experiments. EA developed the genetic lines, performed the experiments, collected and analyzed data. EA and DS wrote the paper with input by AB.

## Electronic supplementary material

DNA markers used to genotype and select chromosome 9 sub-NILs for high-resolution mapping of QTL *stm9*. Marker name and primers used for sequencing of *S. lycopersicum* cv. T5 and interspecific F_1_ hybrid to identify SNP polymorphisms are listed. Sequenom primers for amplification of target polymorphisms, extension primer (specific to polymorphism) and SNP polymorphism detected are given. For markers in which multiple SNPs were genotyped, additional rows are added for extension primers and their corresponding SNP polymorphisms. The Origin column refers to the source of the marker: Established (available at SGN), or New to This Study. (PDF 16 kb)

List of annotated genes downloaded from the *S. lycopersicum* Gene Track from ITAG release 2.40 (SGN solgenomics.net) that are located within the syntenic region containing QTL *stm9*. Genes are listed in order along the short arm of chromosome 9 (from telomere end towards the centromere), from marker H358 to T1673. Gene ontology (GO) terms and InterPro (IPRO) protein domains associated with each gene are listed. (PDF 97 kb)
