# Supplementary material for: High-resolution mapping of a major effect QTL from wild tomato Solanum habrochaites that influences water relations under root chilling
Source: Theor Appl Genet. 2015 Jun 5;128(9):1713–24. doi: 10.1007/s00122-015-2540-y (PMC4540768; doi:10.1007/s00122-015-2540-y)
Supplement: Supplementary file 1 — DNA markers used to genotype and select chromosome 9 sub-NILs for high-resolution mapping of QTL stm9. Marker name and primers used for sequencing of S. lycopersicum cv. T5 and interspecific F1 hybrid to identify SNP polymorphisms are listed. Sequenom primers for amplification of target polymorphisms, extension primer (specific to polymorphism) and SNP polymorphism detected are given. For markers in which multiple SNPs were genotyped, additional rows are added for extension primers and their corresponding SNP polymorphisms. The Origin column refers to the source of the marker: Established (available at SGN), or New to This Study. (PDF 16 kb) [file 122_2015_2540_MOESM1_ESM.pdf]

High-resolution mapping of a major effect QTL from wild tomato *Solanum habrochaites* that influences water relations under root chilling

Theoretical and Applied Genetics

Erin M. Arms, Arnold J. Bloom and Dina A. St.Clair

Corresponding Author: Erin M. Arms emarms@ucdavis.edu
University of California-Davis: Plant Sciences Department

		Sequencing Primers		Sequenom Primers			
**Marker**	**Origin**	**Forward**	**Reverse**	**Forward**	**Reverse**	**Extension Primers**	**Polymorphism**
**T1670**	Established--CAP	ATTCAAGTGGAACCAATACATGG	AATCATGCAGCACTTGGAATATC	ACGTTGGATGGGCCTGTTTACTTTCCATAAG	ACGTTGGATGTTCAAAAGGAAACAGAAGC	TACTTTCCATAAGTTTCTCCAAT	[T/G]
**TG18**	Established--CAP	AAGGGTTGTTGATTCCGTCA	GCACCAGGTTTTCCATCTGT	ACGTTGGATGCCTGGAAATGGTAGGTTAAG	ACGTTGGATGATGGAAAGAGAAGTGATGGC	TCTTTGCTGTTCTGTCATTAA	[T/C]
**H9**	New This Study	TCATGAACACCTTCCTGTGC	GAATCACCTACATCCATTCTGC	ACGTTGGATGGCAACAATTCTGTAATACCC	ACGTTGGATGTTTTTAGCTACACTACCAG	ctggAATACCCTCAGGCAATAG	[A/C]
						gcAAGAAACAACAGTTTAACTTTGA	[C/G]
**H358**	New This Study	TAAGGCCAACAACTTTAACC	ACTTGTCGATCAACGTAACC	ACGTTGGATGTTTCACATTTTCTTGAACC	ACGTTGGATGGCGTCGTCCTGTATATTTCG	GAAATGAGTAAAGAATCAAGCTA	[A/G]
**H348**	New This Study	GTTGTTCACGAATGGATACC	ACATGCCAAAGTTTGTTACC	ACGTTGGATGGGGAATCGGTTAAACTCAAG	ACGTTGGATGGGAGATGGCCTTATATTTTG	TTGCTTTGAAAGGAAATAGT	[G/A]
						ggATTTTGTCTGATTGACAGTC	[A/G]
**At5g11560**	Established--CAP	TGTAGCAGCGGTTCAGAGAA	TAGCTTGCGTGTGAACAACC	ACGTTGGATGTTGTACTTACACGAGCAGGG	ACGTTGGATGAGCATTCAGTAGTCGAGACC	AGCAGGGAAACTTTTTGC	[A/T]
						tcCCAAACAATTCGGCCATC	[T/A]
**T1673**	Established--SCAR	CTAATTTTATTGAAGCCTCAGATGG	AAATCATCAACTCATCCACCATAG	ACGTTGGATGGTTTTGCCATGACAAGTTTG	ACGTTGGATGGAGAGAAGAAGAGTGATGTA	TGACAAGTTTGGTTAACAAT	[G/A]
**H14**	New This Study	GTGAACTTGACGAGAGAAGC	AGTGGGACACTAAAACATCG	ACGTTGGATGGTGTGACCATCTCAAAAATAA	ACGTTGGATGCTCATTTAATTGGATTCTC	cTGGTTGACAATCGGTC	[C/T]
						AACCATTTCGGTTTCATC	[C/T]
**T0532**	Established--SCAR	AAGGTTAACCAAATCGGTAGTGTG	AGTCTGCTTGTTATTTCACCAAGG	ACGTTGGATGTAATGACCAGTCACCGCAGG	ACGTTGGATGATTAGAACATGACTACTCG	CACCGCAGGTAAAGAA	[C/T]
						CCTCATCCTCCACGATA	[T/A]
**TG223**	Established--SCAR	CAAGAAAATATTGTGTAGTGTTCTCCA	TCCCCCTCTTCATCAAATTC	ACGTTGGATGGAATTTCCTGTCACTGTTCC	ACGTTGGATGGTGGTTATGACGCTATGGTG	CTGTTCCTAAGGAACAATAATTCT	[G/A]
				ACGTTGGATGTTACCATCTGGAGCTTGAGG	ACGTTGGATGAGAAGACTTTTTGGATTAG	TAGTCCCTCTTCTGAACAT	[G/A]
