# Supplementary material for: High-resolution mapping of a major effect QTL from wild tomato Solanum habrochaites that influences water relations under root chilling
Source: Theor Appl Genet. 2015 Jun 5;128(9):1713–24. doi: 10.1007/s00122-015-2540-y (PMC4540768; doi:10.1007/s00122-015-2540-y)
Supplement: Supplementary file 2 — List of annotated genes downloaded from the S. lycopersicum Gene Track from ITAG release 2.40 (SGN solgenomics.net) that are located within the syntenic region containing QTL stm9. Genes are listed in order along the short arm of chromosome 9 (from telomere end towards the centromere), from marker H358 to T1673. Gene ontology (GO) terms and InterPro (IPRO) protein domains associated with each gene are listed. (PDF 97 kb) [file 122_2015_2540_MOESM2_ESM.pdf]

High-resolution mapping of a major effect QTL from wild tomato *Solanum habrochaites* that influences water relations under root chilling
Theoretical and Applied Genetics

Erin M. Arms, Arnold J. Bloom and Dina A. St.Clair

Corresponding Author: Erin M. Arms emarms@ucdavis.edu
University of California-Davis: Plant Sciences Department

Gene	Location	Description	GO term(s)	IPRO Domain(s)
Solyc09g008310.2	SL2.40ch09:1772595..1757098	CinA-like protein<br>(AHRD V1 *--- CINAL_BACFN)	GO:0008152 – metabolic process<br>GO:0006777 – Mo-molybdopterin cofactor biosynthetic process	IPR002500 Phosphoadenosine phosphosulphate reductase
Solyc09g008320.2	SL2.40ch09:1783996..1786766	Xyloglucan endotransglucosylase/hydrolase 12<br>(AHRD V1 **** C0IRH1_9ERIC)	GO:0016798 – hydrolase activity, acting on glycosyl bonds<br>GO:0016762 – xyloglucan:xyloglucosyl transferase activity	IPR016455 Xyloglucan endotransglucosylase/hydrolase
Solyc09g008330.2	SL2.40ch09:1803879..1794415	E3 ubiquitin-protein ligase RFWD3<br>(AHRD V1 *--- RFWD3_HUMAN)	GO:0008270 – zinc ion binding	IPR001841 Zinc finger, RING-type
Solyc09g008340.2	SL2.40ch09:1804838..1809925	F12E4.230-like protein (Fragment)<br>(AHRD V1 ***- Q5YJQ6_HYAOR)	None	None
Solyc09g008350.2	SL2.40ch09:1817453..1822095	Amino acid binding protein<br>(AHRD V1 **-- B6TMA2_MAIZE)	GO:0008152 – metabolic process	IPR002912 Amino acid-binding ACT
Solyc09g008360.2	SL2.40ch09:1828731..1823490	Heparan-alpha-glucosaminide N-acetyltransferase<br>(AHRD V1 *--- D2QUR2_SPILD)	None	None
Solyc09g008370.1	SL2.40ch09:1835385..1837232	Nodulin-like protein (Fragment)<br>(AHRD V1 ***- O81121_ARATH)	None	IPR010658 Nodulin-like
Solyc09g008380.2	SL2.40ch09:1844382..1849775	Pectate lyase-like protein<br>(AHRD V1 **** Q56XU8_ARATH)	GO:0016829 – lyase activity	IPR002022 Pectate lyase/Amb allergen
Solyc09g008390	SL2.40ch09:1853126..1852311	MYB transcription factor<br>(AHRD V1 **** Q56UT4_ORYSJ)	GO:0005515 – protein binding<br>GO:0003700 – sequence-specific DNA binding transcription factor activity	IPR015495 Myb transcription factor
Solyc09g008400.2	SL2.40ch09:1859965..1854557	Serine/threonine-protein phosphatase<br>2A 56 kDa regulatory subunit delta isoform<br>(AHRD V1 ***- 2A5D_HUMAN)	GO:0008266 – poly(U) RNA binding<br>GO:0019901 – protein kinase binding	IPR002554 Protein phosphatase 2A, regulatory B subunit, B56
Solyc09g008410.2	SL2.40ch09:1869636..1861145	Peptidyl-prolyl cis-trans isomerase<br>(AHRD V1 ***- D8SL30_SELML)	GO:0051082 – unfolded protein binding	IPR002130 Peptidyl-prolyl cis-trans isomerase, cyclophilin-type
Solyc09g008420.2	SL2.40ch09:1871903..1876525	Carbonic anhydrase (Carbonate dehydratase)<br>(AHRD V1 **** C1DTU5_SULAA)	GO:0004089 – carbonate dehydratase activity	IPR018340 Carbonic anhydrase, CAH1-like
Solyc09g008430.2	SL2.40ch09:1881169..1876963	CHY zinc finger family protein expressed<br>(AHRD V1 ***- Q10RT9_ORYSJ)	GO:0005102 – receptor binding	
Solyc09g008440.1	SL2.40ch09:1894187..1895119	Zinc finger family protein<br>(AHRD V1 ***- D7LWQ5_ARALY)	GO:0003700 – sequence-specific DNA binding transcription factor activity	IPR007087 Zinc finger, C2H2-type
Solyc09g008450.2	SL2.40ch09:1901483..1905512	Ankyrin repeat domain protein<br>(AHRD V1 **-- C0FAG4_9RICK)	GO:0005200 – structural constituent of cytoskeleton	IPR002110 Ankyrin
Solyc09g008460.2	SL2.40ch09:1911197..1905656	Ras-related protein Rab-18<br>(AHRD V1 ***- B6TSE5_MAIZE)	GO:0015031 – protein transport<br>GO:0007264 – small GTPase mediated signal transduction<br>GO:0005525 – GTP binding<br>GO:0007165 – signal transduction<br>GO:0005622 – intracellular	IPR015598 Rab18
Solyc09g008470.2	SL2.40ch09:1916797..1921294	Splicing factor 3a subunit 2<br>(AHRD V1 ***- D8U746_VOLCA)	GO:0005634 – nucleus<br>GO:0008270 – zinc ion binding	IPR000690 Zinc finger, C2H2-type matrin
Solyc09g008480.2	SL2.40ch09:1929572..1921241	Phosphatidylinositol-4-phosphate 5-kinase 9<br>(AHRD V1 ***- B6SXI7_MAIZE)	GO:0005515 – protein binding	IPR017163 Phosphatidylinositol-4-phosphate 5-kinase, plant
Solyc09g008490.2	SL2.40ch09:1945871..1983208	HEAT repeat family protein expressed<br>(AHRD V1 *-*- Q10RT3_ORYSJ)	GO:0005488 – binding	IPR011989 Armadillo-like helical
Solyc09g008500.1	SL2.40ch09:1985345..1984459	Non-specific lipid-transfer protein<br>(AHRD V1 ***- Q1PCI0_SOLCH)	GO:0005516 – calmodulin binding	IPR013770 Plant lipid transfer protein and hydrophobic protein, helical
Solyc09g008510.1	SL2.40ch09:1997493..1996000	UDP-glucosyltransferase family 1 protein<br>(AHRD V1 **** C6KI43_CITSI)	GO:0080046 – quercetin 4'-O-glucosyltransferase activity<br>GO:0080044 – quercetin 7-O-glucosyltransferase activity	IPR002213 UDP-glucuronosyl/UDP-glucosyltransferase
Solyc09g008520.2	SL2.40ch09:2012302..2003633	Chromodomain helicase DNA binding protein 3 (Fragment)<br>(AHRD V1 *--* B1AR16_MOUSE)	GO:0003677 – DNA binding	IPR019787 Zinc finger, PHD-finger
Solyc09g008530.1	SL2.40ch09:2027165..2020056	RRP1<br>(AHRD V1 *--- B3RH41_MEDTR)	None	IPR009053 Prefoldin
Solyc09g008540.1	SL2.40ch09:2033464..2034365	Acetyl xylan esterase A<br>(AHRD V1 *--- D0TLQ4_9BACE)	None	IPR005181 Protein of unknown function DUF303, acetylesterase putative
Solyc09g008550.2	SL2.40ch09:2036208..2039695	NCS1 family transporter cytosine/purines/uracil/thiamine/allantoin<br>(AHRD V1 *--- A4RZF3_OSTLU)	GO:0016020 – membrane	IPR001248 Permease, cytosine/purines, uracil, thiamine, allantoin
Solyc09g008560.2	SL2.40ch09:2048866..2052899	1-aminocyclopropane-1-carboxylate oxidase<br>(AHRD V1 **-- ACCO_MUSAC)	GO:0016706 – oxidoreductase activity, acting on paired donors, with incorporation or reduction of molecular oxygen<br>GO:0016682 – oxidoreductase activity, acting on diphenols and related substances as donors, oxygen as acceptor	IPR005123 Oxoglutarate and iron-dependent oxygenase
Solyc09g008590.2	SL2.40ch09:2057510..2056511	Unknown Protein<br>(AHRD V1)	None	None
